# Noteworthy perspectives on microglia in neuropsychiatric disorders

**DOI:** 10.1186/s12974-023-02901-y

**Published:** 2023-10-04

**Authors:** Hongrui Zhu, Ao Guan, Jiayuan Liu, Li Peng, Zhi Zhang, Sheng Wang

**Affiliations:** 1 Department of Anesthesiology, First Affiliated Hospital of USTC, Division of Life Sciences and Medicine, University of Science and Technology of China, Hefei, 230001 Anhui China; 2 Hefei National Laboratory for Physical Sciences at the Microscale, Division of Life Sciences and Medicine, University of Science and Technology of China, Hefei, 230001 Anhui China; 3 School of Medicine, Xiamen University, Xiamen, 361102 China

**Keywords:** Microglia, Homeostasis, Heterogeneity, Neuropsychiatric disorder

## Abstract

Microglia are so versatile that they not only provide immune surveillance for central nervous system, but participate in neural circuitry development, brain blood vessels formation, blood–brain barrier architecture, and intriguingly, the regulation of emotions and behaviors. Microglia have a profound impact on neuronal survival, brain wiring and synaptic plasticity. As professional phagocytic cells in the brain, they remove dead cell debris and neurotoxic agents via an elaborate mechanism. The functional profile of microglia varies considerately depending on age, gender, disease context and other internal or external environmental factors. Numerous studies have demonstrated a pivotal involvement of microglia in neuropsychiatric disorders, including negative affection, social deficit, compulsive behavior, fear memory, pain and other symptoms associated with major depression disorder, anxiety disorder, autism spectrum disorder and schizophrenia. In this review, we summarized the latest discoveries regarding microglial ontogeny, cell subtypes or state spectrum, biological functions and mechanistic underpinnings of emotional and behavioral disorders. Furthermore, we highlight the potential of microglia-targeted therapies of neuropsychiatric disorders, and propose outstanding questions to be addressed in future research of human microglia.

## Background

Microglia, as the only resident cells of mononuclear–phagocyte lineage in the brain parenchyma, account for approximately 5–10% of the central nervous system (CNS) cells in mice and 0.5–16.6% in humans, with a higher density in white matter than grey matter [1, 2]. Since the advent of sequencing and imaging techniques, ontogeny of microglia has been deciphered from hematopoietic progenitors, macrophages, early microglia, pre-microglia to adult microglia, as well as their turnover with aging [2–6]. The gene profile of microglia in most mammals are relatively conservative, while human microglia exhibit extensive heterogeneity that predominantly enriched in host defense and immune response modulation [7–10]. Mature microglia within the parenchyma cannot be sustained by circulating blood cells; instead, it is solely dependent on their self-renewal coupled with proliferation and apoptosis, enabling a whole repopulation of microglia once about every 100 days [2, 11]. Specially, retinal microglia are repopulated not only from residual microglia but also from macrophages in the ciliary body and iris [12]. The development, maturation and maintenance of microglia are regulated by complex signals, such as CSF1R, RUNX1, CX3CR1, TGF-β, IL-34, etc*.* [13–17]. Neurons and astrocytes also collaborate to preserve microglial ramified morphology and homeostatic gene signature [18–20].

Throughout lifespan, microglia perform pleiotropic biological functions through intricate interactions with other cells, including neurons, astrocytes, oligodendrocytes endothelial cells, T cells, etc*.* by either chemical signals or direct contact. Microglial dysfunction in these processes can lead to persistent disturbance in neurogenesis, blood–brain barrier (BBB) integrity, immune homeostasis and synaptic plasticity [21]. Other emerging discoveries of microglia, such as innate immune memory [22], self-renewal [11] and durotaxis (mechanosensing) [23], have revealed the multifaced role of microglia in different (patho)physiological conditions. As immunosensors, microglia possess abundant receptor repertoire binding with threat-induced endocrine hormones (e.g., glucocorticoids), immune mediators (e.g., DAMPs, IL-1), neural transmitters (e.g., norepinephrine, 5-HT); in turn, they release various immunological factors to impinge neuro-immune microenvironment [24]. As neural modulators, microglial impairment in neurogenesis, neuronal activity and synaptic remodeling leads to neural maladaptation during stress exposure [25], all of which puts microglia in the unique position linking biological or social stress with emotional and behavioral consequences. Based on mastering the advances in microglial biology, we further systematically discuss the notion that microglia play a critical role in the regulation of emotion and behavior, and explain how fetal exposure, lifestyle and other internal or external environmental stress impact microglia functional states, leading to the vulnerability or progression of neuropsychiatric disorders.

## Microglial diversity and state spectrum

Microglia are distributed around neuronal somas, axons, dendrites and blood vessels, exhibiting spatially and temporally restricted profiles determined by both genetic and environmental factors [26–30]. Since microglia classification has entered an exquisite phase with single-cell sequencing (scRNA-seq), the dichotomized M1/M2 polarization framework has been gradually discarded [10, 31–34]. Here, microglial “subtypes” are described by the relatively stable molecular and functional profiles, while a vast landscape of microglial “states” is distinguished corresponding to different pathologies, disease stages, or even culture conditions.

### Keratan sulfate proteoglycan (KSPG) microglia

A subpopulation with abundant expression of KSPG has a preferential distribution in the hippocampus, brainstem, olfactory bulb (OB), spinal cord and retina, though relatively little is understood about their function [10].

### Hox8b microglia

Hox8b microglia is much lesser than non-Hoxb8 microglia, but their loss cannot be compensated by the latter [35]. This subtype is associated with abnormal behaviors. Ablation of Hox8b microglia can induce obsessive–compulsive disorder (OCD)-like and anxiety-like behaviors in mice [36–38]. However, optogenetic stimulation of Hox8b microglia in specific brain areas results in enhanced anxiety, compulsive grooming, or both abnormalities [39].

### TREM2 microglia

In the brain, TREM2 is exclusively expressed by microglia regulating their survival, proliferation, metabolism and phagocytosis [40]. Whereas, not all microglia express TREM2, as demonstrated by the region-specific distribution in murine brain [41]. TREM2 microglia has been intensively investigated in response to amyloid β (Aβ) and tau [40, 42, 43], while recent studies revealed distinct responses of cortical and subcortical TREM2 microglia to the Alzheimer’s disease (AD) pathology [44]. This subpopulation also participates in TDP-43-associated neurodegeneration [45] and axonal injury repair [46].

### CD11c microglia

This subpopulation is the major source of insulin-like growth factor 1 (IGF-1) that is vital to myelinogenesis and neurodevelopment [47, 48]. Although CD11c^+^ microglia, proliferative region-associated microglia (PAM), and axon tract-associated microglia (ATM) are described by different terms, they may represent the same population referred as “developmental CD11c^+^ microglia” [47, 49, 50]. During neurodegenerative settings, microglia are transformed into DAM, activated microglia, or microglial neurodegenerative phenotype (MGnD) associated with the brain pathology, all of which exhibit an upregulation of CD11c [48, 51–53].

### Arginase-1 (ARG1) microglia

In physiological condition, ARG1 microglia is enriched in the basal forebrain and ventral striatum during early development, displaying upregulation of *Apoe, Clec7a, Igf1, Mgl2* and *Lgals3* compared to ARG1 negative microglia. This subpopulation plays a pivotal role in shaping cholinergic forebrain–hippocampus circuits involved in cognitive function [54]. ARG1-expressing microglia induced by IL-4 appear to be neuroprotective by restoring hippocampal neurogenesis associated with stress resistance [55] and enhancing Aβ clearance in the context of neuroinflammation [56].

### Microglia supporting neurogenesis

In active neurogenetic niches, including OB, subventricular zone and rostral migratory stream, microglia can be TREM2-negative and Iba1-negative, and they persist beyond the ontogenetic period [57, 58]. In the subgranular zone of dentate gyrus, microglia expressing Clec7a is correlated with adult neurogenesis [49].

### Repopulating microglia

Repopulating microglia cells demonstrate enhanced cell-cycle, migration and survival-related genes to support their rapid replenishment [11]. However, substantial microgliosis is only observed in the gray matter following diphtheria toxin-induced ablation but not in the white matter, indicating the regional heterogeneity of microglia repopulation [59].

### Lipid droplet accumulating microglia (LDAMs)

This notion emerges from the “foamy macrophages” in atherosclerosis. These glial cells containing massive lipid droplets were found in the brains of aging [60], AD [61, 62], and diabetes-associated cognitive impairment [63], displaying pro-inflammatory states and phagocytic defects.

### Microglia in neurodegeneration

Various microglia states were identified under different neurodegenerative contexts. Disease-associated microglia (DAMs) were first described as a protective phagocytic microglia population in AD, generated from homeostatic microglia through a two-step TREM2-independent and -dependent mechanism [64, 65]. Yet, proinflammatory DAMs driven by receptor-interacting protein kinase 1 (RIPK1) also existed and were deleterious in the etiology of AD [66, 67]. Silvin et al*.* further identified embryonically derived TREM2-dependent DAM with neuroprotective signature and monocyte-derived TREM2-independent disease inflammatory macrophages (DIMs) accumulating in aging brain [68]. Activated response microglia (ARMs) were detected in Aβ (but not tau) models exhibiting upregulation of *Apoe, Clec7a*, MHC class II and putative tissue repair genes, which might be the converging point of AD risk factors [69, 70]. In hippocampal CA1 and temporal cortex, Aβ plaque-associated microglia were positive for all NLRP3 inflammasome components and cleaved gasdermin D, presenting pyroptosis activation in microglia responding to amyloidosis [71]. Interferon response microglia (IRMs) were defined from Aβ models by enhanced interferon type I pathways, including Ifit2, Ifit3, Ifitm3 and Irf7 [70, 72]. They displayed efficiency at restricting Aβ accumulation in AD mice [72], but also existed in young wild-type mice and increased over normal aging [70]. Pathology-specific AD1–microglia subgroups localized to Aβ and AD2–microglia subgroups associated with p-tau [73], as well as temporally specific early stage AD-associated microglia (EADAMs) and late-stage AD-associated microglia (LADAMs) [74], were identified by distinct genetic and functional signatures.

In addition to AD, MGnD was defined across AD, amyotrophic lateral sclerosis (ALS) and multiple sclerosis (MS) associated with neurodegenerative progression [75]. A subtype with a distinct ferroptosis-associated transcriptomic signature was enriched in the spinal cord of ALS and midbrain of Parkinson’s disease (PD) patients [76]. In the nigra of PD patients, microglia demonstrated a proinflammatory phenotype as a part of “pan-glial” activation network [77]. These findings revealed markable plasticity of microglial states, yet whether they are strictly correlated with certain diseases or more universal core properties, and whether they are conserved in humans, still need exploration [34, 43].

### Senescent microglia

Microglial senescence was characterized by less ramified, reduced process motility and increased inflammatory responses to brain injury [78–81]. Age-associated changes in human microglia were enriched in axonal guidance, cell adhesion, actin (dis)assembly and surface receptor expression, and they can engulf the dead neurons in aged brain [8, 82].

### Microglia in vitro

Mature microglia lose their signature (*Tmem119, P2ry12, Sall1*) and dedifferentiate within hours of isolation. Cultured microglia exhibit downregulated inflammation but upregulated cell-cycle and complement-related proteins [16]. Microglial profile in vitro cannot perfectly mimic the situation in vivo, for which stringent experimental conditions (e.g., astrocyte-conditioned medium) are required to maintain microglial homeostasis in vitro [83–86].

## Biological functions of microglia

### Microglia in brain development and neurogenesis

Distinct from prevailing ramified microglia in the gray matter, a proliferative region-associated CD11c^+^ microglia subpopulation displaying amoeboid morphology is discovered in the white matter which phagocytoses newly generated oligodendrocytes or myelin sheath and engages in the myelinogenesis [49, 50]. They are highly efficient at the clearance of apoptotic debris produced by dead cells during brain development [87, 88]. Importantly, trophic support from the CNS cellular milieu is required for neurogenesis. In the developing cortex, microglia-derived IGF-1 is essential for the survival of layer V neurons [89]. These activities also occur in young adults, as the unchallenged microglia in the subgranular zone niche dynamically remodel hippocampal circuitry by phagocytosing apoptotic newborn cells [90]. After traumatic brain injury, repopulating microglia can robustly promote adult neurogenesis in the hippocampus through IL-6/sIL-6R signaling [91].

### Immune surveillance and homeostasis

In healthy brains, microglia provide continuous surveillance for CNS microenvironment by extending and retracting their processes. They are sensitive to pathogen infections, damage-associated molecular patterns (DAMPs) and peripherally produced neurotoxins to maintain CNS homeostasis. Neuronal injury initiates long-range Ca^2+^ transients and ATP release as guidance cues for the migration of P2Y12-expressing microglia [92–95]. However, constant microglial ramification and surveillance are independent of purinergic signaling. Instead, it requires the tonic activity of several channels, such as TWIK and TRPV1 [96–98]. Another aspect worth considering is that both immune surveillance and response are highly energy-demanding processes, which are coupled to metabolic adaptations, such as glycolytic flux and mitochondrial activity in microglia [99]. Microglia participate in antiviral immune by initiating CD4 + T cells responses and cross-presenting antigen to CD8 + T cells that remove the infected neurons by noncytolytic mechanism [100–102]. At the early stage of viral infection, phosphatidylserine (PtdSer) serves as a “eat-me” signaling recognized by microglial TAM receptors and triggers the phagocytosis of compromised cells [94, 95]. Candida infection in the parenchyma evokes focal microgliosis and astrocytosis surrounding the fungal cells to form fungal-induced glial granuloma (FIGG), in which microglia release cytokines, such as IL-1β, IL-6, CXCL1 and TNF to enhance fungal clearance [103, 104]. Several non-infectious CNS diseases are also characterized by neuroinflammation driven by microglial sensors or pathways, such as RIPK1, interferon-induced protein 35 (IFP35) and Toll-like receptors (TLRs) [105–107].

### Microglia regulate vascular architecture and cerebral blood flow

Microglial regulation of angiogenesis was corroborated in the retinas of mice and zebrafish, as they promote vascular anastomosis during developmental neovascularization via an ambiguous TGF-β-dependent but VEGFA-independent mechanism [108–110]. In adults, microglia retain the capability to regulate endothelial function in the presence of chronic activation of TGF-β signaling. For example, obesity can elicit microgliosis and activation of microglial TGF-β-activated kinase 1 (Tak1), which further increases the secretion of IL-18 that directly inhibits the activity of endothelial nitric oxide synthase (eNOS) and hence eliminates NO production, leading to cerebrovascular dysfunction [111]. Low oxygen concentration is another driving force for neovascularization, which is partly related to the recruitment of microglia towards the sites of endothelial cell anastomosis induced by MAS receptor (encoded by *Mas1* gene) signaling [112]. He et al. reported a proangiogenic receptor-interacting protein 3 positive (RIP3^+^) microglia subpopulation that can be activated by hypoxia to release fibroblast growth factor 2 (FGF2) through RIP3-mediated necroptosis and associated with retinopathy [113]. Capillary-associated microglia (CAMs) are the bona fide microglia residing in the perivascular space. They have direct purinergic interactions with endothelium and periarterial smooth muscle cells, contributing to the maintenance of optimal capillary radius, vascular reactivity and cerebral blood flow, which may be particularly crucial in pathological conditions, such as hypoperfusion and hypercapnia [114, 115].

### Microglia affect BBB permeability

Being a component of the neurovascular unit (NVU) and glial–vascular unit (GVU), ongoing studies indicate a critical role of microglia in the disruption of perivascular niche and BBB integrity [116, 117]. Microglia activated by ischemic stroke can secrete TNF, adipocyte fatty acid-binding protein (A-FABP) and metalloproteinase 9 (MMP-9) to trigger endothelial necroptosis and BBB leakage [118–120]. Research from Haruwaka and colleagues described the time-dependent dual effects of microglia on BBB permeability. Microglia initially migrate towards the cerebral blood vessels driven by CCR5 and protect BBB integrity by producing tight-junction protein Claudin-5 (CLDN5) and physically interacting with the endothelium. However, persistent inflammation boosts these vessel-associated microglia to engulf astrocytic end-feet and wreck BBB function [121].

### Microglia as sculptors of synaptic plasticity

Microglia interact with synaptic structures throughout the lifespan, particularly in the first two postnatal weeks. During early development, microglial TREM2 critically controls the refinement of excessive synapses [122]. P2Y12 expressed on microglia is required for synaptic remodeling in the developing visual cortex, indicating the contribution of ATP to this process [123]. Complement system (C1q, CR3, CR5) is well-known to mediate microglial pruning and engulfment of synapses [124–126]. Yet in the barrel cortex, synapse elimination is CR3-independent but regulated by CX3CR1 [127]. Cytokine IL-33, a novel regulator of microglial activation, can be secreted by developing astrocytes or adult hippocampal neurons and promote microglia-dependent synapse remodeling under physiological conditions [128, 129]. Serotonin (5-HT) is another important signaling directing microglial pruning during neonatal phase. Impaired 5-HT sensing reduced phagolysosomal compartments in microglia and synapse–microglia proximity, leading to impaired synaptic and axonal sculpting in the hippocampus, cortex and thalamus [130]. In contrast, SIRPα–CD47 functions as “don’t eat me” signal to prevent excessive microglial engulfment for the maintenance of synaptic structural and functional homeostasis [131]. Recent work by Zhong et al. found the formation of TREM2–C1q complexes in human AD brains; in murine AD models, microglial TREM2 can bind with C1q to rescue synaptic loss [132]. The question remains as how distinct pathways converge or antagonize their effects on lifelong synaptic modeling, as well as how microglia interact with other cells, such as astrocytes and oligodendrocytes to modulate these processes.

### Microglial phagocytosis and efferocytosis

Microglia can phagocytose surviving or dead cells, a three-step process characterized by recognition, phagosome formation and ingestion. During the development of cerebellum, microglia promote the phagocytosis-mediated death of Purkinje fibers and clearance of apoptotic cells induced by phagocytosis-related genes epigenetically restricted by polycomb repressive complex 2 (PRC2) [27]; in the hippocampus, microglial CD11b and DAP12 are involved in this process [133].

The mechanism whereby microglia scavenge cell debris is related to numerous candidate molecules, such as P2Y12, BAI1, TIM-4, TAM receptors (Tyro3, Axl, MerTK), CD22, CD36, SYK, and TREM2. These receptors act at different levels, including recognition, target biding, and phagosome formation [29, 134–141]. Receptor recognition launches signaling to form the phagocytic cups, and then phagosomes are formed and gradually mature by fusion and fission with endosomes and lysosomes endowed with essential molecular markers, such as Lamp1, Rab5 and Rab7 [142–144]. With the assistance of Slc37a2, hybrid vesicles (phagolysosomes) are formed and fused into “gastrosome” containing membrane fragments and cellular debris, which facilitates the cargo degradation by digestive enzymes, such as Cathepsins [145–147]. Insufficient microglial clearance of cell debris and neurotoxic agents plays an important role in the development of diseases, including emotional disorders, stroke, aging and AD [134–136, 138, 141].

### Interactions between microglia and other CNS or peripheral cells

Microglia constantly sense and respond to different signals to ensure neuronal activity under tight check [148]. In physiological conditions, direct microglia–neuron communication exist at the membrane-to-membrane junctions between microglial P2Y12-expressing processes and neuronal somatic Kv2.1-clustering regions, located in the vicinity of neuronal mitochondria and coupled with neuronal status switch [149]. Node of Ranvier is a major site of direct microglia–neuron interaction associated with myelin regeneration [150]. Local mediators, such as purinergic, serotonergic and complement signaling have been widely investigated in microglia–neuron crosstalk across physiological or disease-specific contexts [151–153]. Recent studies addressed microglia as the “brake” to dampen neuronal activity. In the striatum, microglia inhibit D1 neuronal activation via an ATP/AMP/ADO/A1R-dependent negative feedback, which is critical to prevent seizure vulnerability [154]. They also constitutively release platelet-derived growth factor B (PDGFB) that promotes the expression of Kv4.3 channels on neurons to increase neuronal potassium currents, while disruption of this pathway elevates basal sympathetic tonicity and predisposes hypertension [155]. In the thalamic reticular nucleus (TRN), microglia send circadian ceramide signaling projecting at neurons to modulate wake–sleep transition [156].

Glia–glia interactions represent a delicate balance affecting neural functions in health and diseases [157]. Inflammatory mediators and mitochondrial fragments from microglia can convert astrocytes to a neurotoxic A1 phenotype that occurs in many neurological diseases [158–160]. Conversely, microglia-derived TGF-α acts via ErbB1 in astrocytes to limit their neurotoxic activities. In demyelinating diseases, microglial extracellular vesicles release multimodal and multitarget signaling mediators acting on astrocytes and oligodendrocyte precursor cells (OPCs) around myelin lesions [161]. AD pathology drives microglia to express IL-3Rα that renders them responsive to IL-3 constitutively produced by astrocytes, which endows microglia with an acute immune response and recruitment towards Aβ plaque and neurofibrillary p-tau [162].

Cell-to-cell transfer is an interesting process, whereby microglia facilitate either the clearance or propagation of pathological factors. The F-actin-dependent membranous tubular connections between microglia enable them to share the α-synuclein (α-syn) burden for effective degradation, accompanied by the donation of intact mitochondria from naïve to affected microglia to attenuate inflammatory reactions [163, 164]. Burden transfer also occurs between microglia and neurons, yet it may be deleterious, since microglia act as the carriers of Aβ migrating towards unaffected neurons [165].

Microglia interactions with peripheral immune cells were studied in infectious and autoimmune encephalopathy. Upon recovery from neurotropic flaviviruses infection, IFN-γ derived from CD8^+^ T cell provokes microglial activation and excessive synapse elimination, contributing to post-infectious cognitive sequelae [102]. Conversely, CNS immune microenvironment re-established by microglia can also be the trigger for T cell-associated neuroinflammatory diseases [102, 166, 167]. In addition, regulatory T cells (Treg)–microglia interaction enhances the reparative activity of microglia, subsequently promoting oligodendrogenesis and white matter repair after stroke [168]. Lymphocyte–microglia–astrocyte crosstalk was described in chronic MS, in which C1q-activated microglia inflamed in MS (MIMS) and astrocytes inflamed in MS form a central hub to connect with other immune clusters [169]. In AD pathogenesis, meningeal lymphatic drainage is coupled with microglial activation and neurovascular response [170]. Together, interaction between microglia and other cells is ubiquitous at both physiological and pathological states; therefore, microglia dysfunction may have a domino effect on the entire CNS cellular network which demands further study.

### Microglial priming and innate immune memory

Innate immune memory refers to that microglia exposed to a priming or desensitizing stimulus can react stronger (training) or weaker (tolerance) to the subsequent inflammatory insult [171, 172]. This has been observed in stress [173], aging [174, 175], optic nerve crush [176], MS [177], PD [178]and AD [179, 180]. Pregnant or neonatal mice that undergone inflammatory stimuli exhibit altered microglial responses upon a secondary disturbance during adulthood [181–184]. Early postnatal exposure to LPS primes microglial susceptibility to stress at adolescence, which enhances microglial engulfment of glutamatergic neuronal spines and depressive symptoms [185]. However, maternal immune activation (MIA) microglia in the offspring demonstrates a long-lasting decrease in immune reactivity (blunting) across the developmental trajectory [186]. In “tolerant” or “trained” microglia, the first stimulus endows them with epigenetic reprogramming that persists for at least 6 months [22, 187]. LPS-induced microglia priming is intimately linked to H3K4me3 and H3K27Ac peaks at promoters and enhancers, whereas the expression of tolerant genes correlated with ATAC and H3K27Ac peaks at enhancers [188]. Cerebral β-amyloidosis is exacerbated by immune training but alleviated by immune tolerance, which is modified by histone deacetylases-1/2 (HDAC1/2) [187].

Microglial neurotoxicity is usually aligned to primed microglia state due to their aggressive inflammatory state, while microglial tolerance provides sustained neuroprotection [189, 190]. However, the cytokine profile and phagocytotic activity of primed microglia should be taken into consideration, given that increased phagocytosis facilitates the clearance of cell debris, and the pleiotropic cytokines (e.g., IFN-β and IL-6) can be important for injury repair [174, 191]. This conclusion should not be made consistently but rather depend on the certain pathologies.

### Microglial durotaxis

**Fig. 1:**
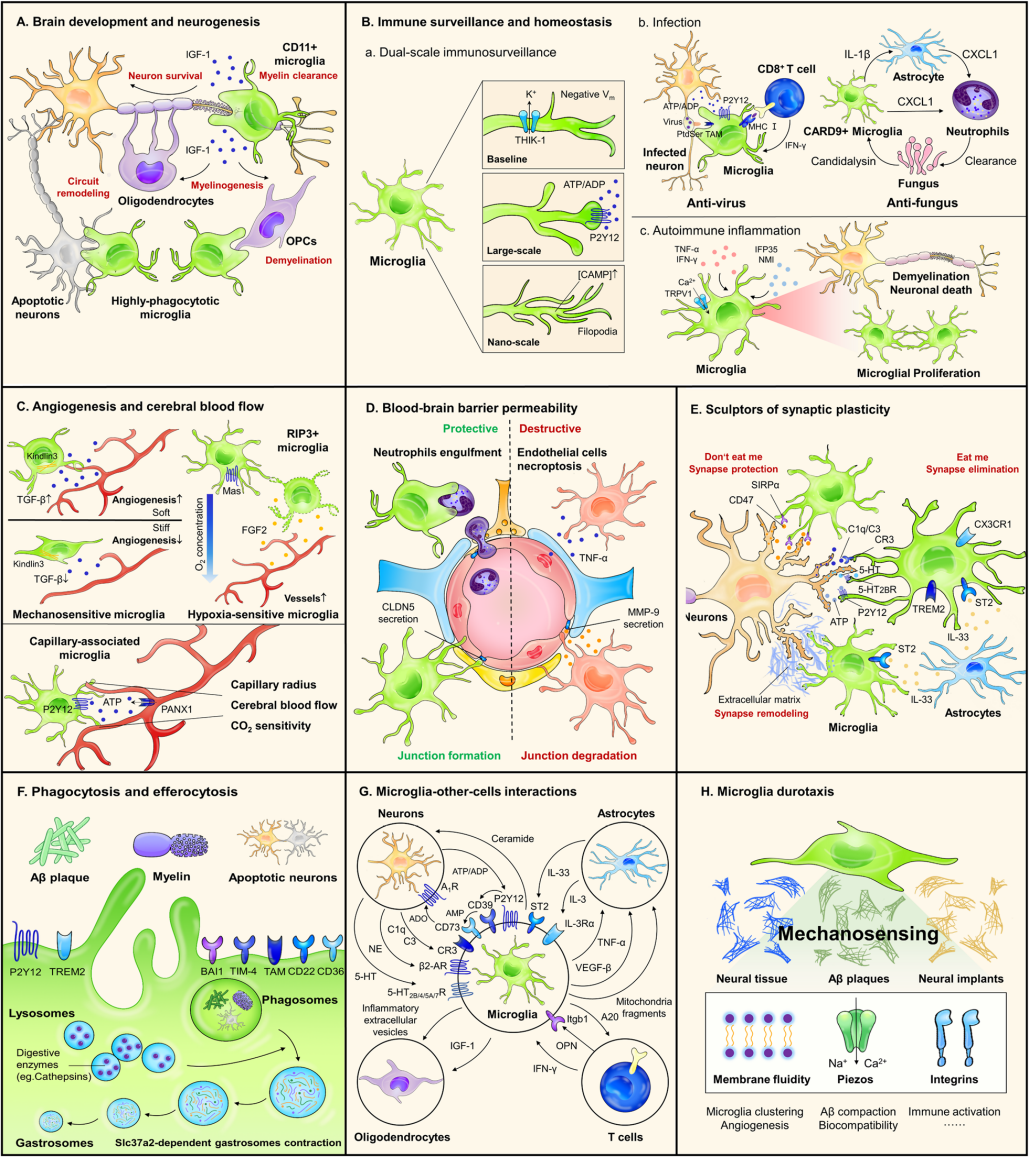
Multiple biological functions of microglia. **A** Microglia in brain development and neurogenesis. CD11c^+^ microglia are critical for neurodevelopment by providing pro-survival IGF-1 signaling for newborn neurons and oligodendrocytes. They also phagocytose apoptotic neurons, excessive myelin sheath and oligodendrocyte precursor cells to accomplish a structure–function equilibrium within neural circuits. **B** Immune surveillance and homeostasis. Microglia perform a dual-scale surveillance of CNS homeostasis by their processes sensitive to environmental perturbations. They are pivotal immune components in CNS infection and autoimmune diseases. **C** Microglia regulate angiogenesis and cerebral blood flow. Tissue stiffness and O_2_ concentration are two important cues for microglia to modulate angiogenesis throughout development. Capillary-associated microglia interact with brain vessels via purinergic signaling to maintain appropriate cerebral blood flow. **D** Dual-role of microglial in BBB integrity. Microglia can rescue BBB damage by secreting claudin 5 and phagocytosing infiltrated neutrophils to ameliorate further brain injury. On the other hand, they also secrete TNF-α to induce degradation of endothelial junctions, which aggravates BBB disruption. **E** Bi-directional role of microglia as sculptors of synaptic plasticity. Except for synaptic elimination via “eat me” signaling, microglia are also guardians of synaptic structures through “don’t eat me” signaling CD47–SIRPα. They engulf extracellular matrix deposition to support the formation and maturation of dendrite spines. **F** Microglial phagocytosis and efferocytosis. Microglia detect and phagocytose apoptotic neurons, myelin sheath and Aβ plaque by different receptors. Inside microglia, the phagosomes undergo hybrid phagolysosomes formation, gastrosomes fusion, and gastrosomes contraction to facilitate the scavenging of debris. **G** Interactions between microglia and other cells. Shared signaling pathways in microglia–other-cells crosstalk include purines, norepinephrine, serotonin, organelle component, complements and cytokines, which exerts profound effect on cellular survival, proliferation, metabolism, and functions. **H** Microglia durotaxis. Microglia react with the mechanical cues from neural tissue, Aβ plaque and foreign implants via mechanosensors, such as Piezos and integrins. The mechanism and significance of microglial durotaxis remain largely unknown

Mechanosensation of environmental cues, including extracellular protein burden and surface rigidity, can directly affect the level of immune response [192–194]. Microglia can sense the mechanical signals in their microenvironment and display a preference to migrate towards stiffer regions, termed as durotaxis [23]. The mechanical cues drive microglial adaptation of cytoskeletal composition and morphology to the surrounding stiffness, presenting spherical morphology with short lamellipodia on soft substrates, but more extended on stiff surface [192]. Multiple families of mechanosensors on microglia are identified [193, 195]. Piezo1 are highly expressed on microglia that can sense the stiffness of Aβ fibrils and engage in the compaction and engulfment of Aβ [196–199]. In retina, Kindlin3 is important for microglial to sense the stiffness of different layers [110] (see Fig. 1).

## Microglia in behavioral and neuropsychiatric disorders

As discussed above, microglia perform a myriad of functions in brain development, synaptic plasticity, neuronal synchronization and neuroimmune homeostasis from embryo to adulthood. For neuropsychiatric disorders presenting emotional and behavioral abnormalities, microglia were initially suggested to reflect an immune response to relevant stress; however, recent genetic analyses revealed that a proportion of genetic risks exerts their impacts through myeloid cells, highlighting a causal role of microglia, the CNS resident myeloid cells, in the neuropsychiatric disorders [200, 201].

### Depression and anxiety disorders

Depression is characterized by persistent sadness, anhedonia and diminished interest accompanied by daily function deficits [202]. Anxiety is arisen excessive fear and anticipation of real or imagined future threat, usually exhibiting avoidance behaviors and physical symptoms [203]. Depression and anxiety share genetic, neurobiological, and psychological risk mechanisms, including monoamine neurotransmission, hypothalamic–pituitary–adrenal axis changes, neuroinflammation, neuroplasticity, neurogenesis, etc*.*, and they are often comorbid and difficult to distinguish, which causes more severe symptoms and treatment difficulties [202–204].

Inflammation is a common biological origin contributing to depression and anxiety. Innate and adaptive immune system dysregulation participate in symptom deterioration and compromise the curative effects of antidepressant and anti-anxiety treatments [205]. Microglia are the key initiators and regulators in CNS inflammatory cascade. Microglia activated by peripheral inflammation medium triggered an IL-6-mediated autocrine loop and the release of prostaglandin E_2_, which decreased the firing of striatal medium spiny neurons via delayed rectifying K^+^-channels, leading to negative affection [206]. In the model of post-stroke anxiety, HDAC3 was upregulated in the microglia of damaged cortex, which induced p65 deacetylation and the subsequent activation of NF-κB signaling involved in prostaglandin synthesis. Prostaglandin E_2_ released by cortical microglia could act on EP2 in the amygdala, potentially leading to the susceptibility to anxiety [207]. Discs large homolog 1 (Dlg1) is an adaptor protein regulating the activation of microglia, and Dlg1 knockout inhibited NF-κB and MAPK pathways in the microglia, leading to the alleviation of LPS-induced depression [208]. Adiponectin (APN), a circulatory hormone secreted by adipocytes, is associated with various neurological disorders. Studies demonstrated that APN deprivation exerted an antidepressant effect through regulating microglia NF-κB/Trk/BDNF signaling [209]. Glucagon-like peptide-1 receptor (GLP-1R) expressed on the microglia is another intriguing but understudied signaling with anti-inflammatory and neurotrophic properties [210]. Activation of GLP-1R ameliorated depression-like behaviors in diabetic db/db mice by promoting mitophagy in hippocampal microglia, which decreased ROS accumulation for final pyroptosis and pro-inflammatory cytokines release from microglia [211]. Interestingly, in animal model of neuropsychiatric systemic lupus erythematosus (NPSLE) that resembling symptoms, such as depression, anxiety and memory deficit, microglia were intrinsically activated in the presence of intact BBB during prenephritic stage, leading to increased IL-6 and IL-18 that elicited apoptosis of adult hippocampal neural stem cells and might gradually impair BBB integrity, indicating microglia as the spark of early neuropsychiatric diseases by altering neuroimmune microenvironment, which initiates BBB disruption and infiltration of peripheral immune components to provoke the disease [212].

An emerging understanding of “behavioral immune system” suggests that psychosocial stress can potentiate mental disorders through immunological and inflammatory pathways [213]. Wu et al. profiled the dorsolateral prefrontal cortex of non-human primates with depressive-like behaviors, showing that genetic alterations associated with depression-like behaviors predominantly enrich in microglia, and a subpopulation of pro-inflammatory microglia of depressive-like phenotypes (PIMID) was identified by upregulation of proinflammatory pathways and psychiatric risk genes, including *BCL2 *and* SYNE1* [214]*.* In repeated social defeat stress (R-SDS) rodent models, IL-1α and TNF-α in mPFC microglia are upregulated in a TLR2/4-dependent manner, which gave rise to neuronal hypo-reactivity, dendritic atrophy and social avoidance [215]. Rather than TLRs, high mobility group box-1 protein (HMGB1) acts via receptor for advanced glycation endproducts (RAGE) on hippocampal microglia and causes anhedonia primed by chronic stress [216]. In the threat appraisal regions of the brain (prelimbic cortex, central amygdala, and hippocampus), R-SDS-induced microglia activation upregulates IL-1R1 on endothelial cells and hence facilitates monocyte recruitment, likely via chemokine gradient CCL2, to promote anxiety-like behaviors [217]. Along with stress accumulation, activation of NLRP3 inflammasome and its downstream inflammatory cytokines in hippocampal microglia contribute to the development of depressive-like behaviors. The mechanisms underlying NLRP3 inflammasome assembly could be associated with the activation of P2X7 receptors and increased serum glucocorticoid level, which activates glucocorticoid receptor (GR)/NF-κB/NLRP3 pathway [218–220]. During chronic mild stress, activation of microglial NLRP3 inflammasome triggers the release of A1 cocktail (TNF-α, IL-1α and C1q) to induce the production of A1-like astrocytes in hippocampus, which begins preceding to the onset of dendritic dysfunction and depression-like behaviors [221].

Microglia dysfunction in synaptic pruning and neuronal circuit modeling has been implicated in the development of depression and anxiety. Early life inflammation can prime microglia to perform enhanced CX3CR1-mediated engulfment of ACC^Glu^ dendritic spines in response to later stress events, which causes the hypoactivity of ACC^Glu^ and depressive vulnerability during adolescence [185]. At adulthood, neuron–microglia communication through CX3CR1–CX3CL1 also affects synaptic plasticity and neurogenesis in hippocampus associated with susceptibility to environmental stress and major depression [222, 223]. Chronic stress increases CSF1 secretion from neurons of mPFC to promote microglial phagocytosis of dendritic spines of pyramidal neurons, leading to depression-like and anxiety-like behaviors [224]. In contrast, early life adversity impairs microglial pruning of excitatory synapses onto stress-sensitive CRH-expressing neurons, provoking hormonal and behavioral stress responses in adulthood [184]. Under normal conditions, synaptic remodeling by microglia in the ventral hippocampal CA1 (vCA1) is active in the daytime, during which ROS and uncoupling protein 2 (UCP2) are produced. Knockout of UCP2 hinders the phased elimination of dendritic spines, resulting in disruption of hippocampal neural circuit and anxiety-like behaviors [225]. Fan et al. found that the exosomes secreted by microglia carrying mir-146a-5p are transported to the dentate gyrus, where they downregulates the expression of Krüppel-like factor 4 (KLF4) and cyclin-dependent kinase-like 5 (CDKL5), inhibits the proliferation and differentiation of neurons, and participates in the onset of depression [226].

Microglia could be the key mediator between negative affection and psychoactive substances, such as alcohol and tobacco. Adolescent ethanol consumption can drive the hyperactivation of microglia in raphe nuclei, leading to increased levels of TNF-α and IL-10, decreased serotonergic activity, and neuropsychiatric consequences of hyperalgesia and depression [227, 228]. Socodato et al. found that alcoholism activates microglia in the mPFC via Src/NF-κB/TNF pathway and selectively enhances the clearance of excitatory synapses, which inhibits nerve conduction and contributes to the anxiety-like behaviors [229]. Microglia depletion by CSF1R inhibitor reduces inhibitory GABA_A_ and excitatory glutamate receptor-mediated transmission in the central nucleus of the amygdala (CeA) and improves alcohol-dependent anxiety [230]. During smoking cessation, increased expression of Nox2 and production of ROS from microglia in the nucleus accumbens potentiate the occurrence of nicotine withdrawal-related anxiety [231].

Although considerable researches have addressed the detrimental impact of microglial activation, the actual microglial role in human depression and anxiety remain largely unknown. Several translocator protein (TSPO) binding positron emission tomography (PET) studies demonstrated enhanced microglial activation in prefrontal cortex (PFC), ACC and insula of patients with major depressive disorder (MDD) [232–234]. However, evidence from post-mortem researches showed elevated homeostatic markers and unchanged or even inhibited inflammatory reactivity of microglia in MDD [37]. A distinct DAM phenotype, termed as DepDAM, was identified from the occipital cortex grey matter of MDD patients, displaying downregulated genes involved in immune response (*C1QA/B/C, SPP1, MK167*) and phagocytosis (*CD14, CD163, FCGR1A/C, FCGR3A*). Acquisition of this immune-suppressed status might be regulated by the increased “don’t eat me” CD200/CD47 signaling between neuron–microglia interaction [235]. A possible explanation is that reduced astrocytes, observed in post-mortem brains of MDD individuals, may cause disruption of BBB integrity and subsequently infiltration of monocytes into the CNS, which can also display increased TSPO in PET [236]; on the other hand, microglia activation may not persist but assume a role at specific stages of MDD [235].

### Autism spectrum disorder (ASD)

ASD is a constellation of neurodevelopmental disorders characterized by early appearing social deficit, repetitive behaviors, restricted interests and sensory abnormality [237]. Hundreds of genes have been identified to decipher the genetic heterogeneity of ASD, pointing towards distinct biological processes, such as protein synthesis, chromatin remodeling, cell adhesion and synaptic function. Transcriptomic analysis provided corroborative evidence of microglial molecular perturbations preferentially in ASD patients [238]. Publication by Ma and colleagues revealed that disruption of microglial homeostasis impaired the supporting microenvironment of neural progenitor cells due to the loss of plasticity-related genes 3 (PRG3) triggering Wnt/β-catenin cascade, leading to abnormal neuronal plasticity during embryonic development and autistic behaviors at later stages [239].

Synaptic abnormality is a major hallmark of ASD, reflected as altered local protein synthesis, spine architecture and signaling transmission, and thus ASD is classified as a “synaptopathy” [240]. During brain development, synapses undergo substantial pruning and engulfment performed by microglia, which are essential for synaptic refinement and neural circuit maturation. It has been widely established that mutations lacking nuclear localization of phosphatase and tensin homolog deleted on chromosome ten (PTEN) are associated with ASD. Sarn et al. showed that cytoplasmic predominant–PTEN localization elicits excessive C1q-mediated synaptic pruning by microglia [241]. Engulfed synapses can be degraded by microglial autophagy, and depletion of autophagy-related gene 7 (Atg7) results in synaptic surplus and subsequent autistic behaviors due to impaired synaptosome degradation in microglia [242]. Patients aged in the developmental window coinciding with synaptic refinement (between 5 and 23 years) exhibits a significant decrease in TREM2 expression relative to healthy individuals. The absence of TREM2 attenuates microglia-mediated synapse elimination in CA1 accompanied by underconnectivity between prefrontal and hippocampal regions, contributing to the social defects and repetitive behavior in *Trem2*^*−/−*^ mice. This study further supports the centrality of microglia in the nervous-immune crosstalk for correct brain development [122]. Among the neurotransmitter systems, serotonin (5-HT) is one of the longest standings in ASD etiology [243]. Microglia express 5-HT_2B_ receptor throughout postnatal lifetime. During a critical developmental window from birth to P30, serotonergic signaling between synapses and microglia is pivotal for synaptic refinement and circuitry maturation involved in sociability and flexibility in novel environment [130]. The deficiency of glutaminase 1, an enzyme responsible for glutamate generation in the brain, can also impair microglial synapse pruning and results in ASD-like behaviors [244].

Fetal environmental exposure, especially MIA during pregnancy, is a long-term risk factor of ASD. Autistic behaviors such as social deficit, repetitiveness and anxiety have been observed in MIA-affected offspring [245–247]. Since prenatal BBB is not mature, maternal cytokines and pathogenic antibodies targeting fetal brain antigens can directly invade fetal CNS through placenta, or indirectly elicit the production of endogenous cytokines in fetus, which has profound impact on immune, neuronal and synaptic homeostasis of the offspring [247]. Human microglia progressively mature from gestational week 13, and by midgestation they have progressed towards homeostatic immune-sensors with the signature profiles of sensome, including *Csf1R, Cx3cr1, Cxcl16, Ifngr1 *and* Ly86*, rendering prenatal brain vulnerable to environmental disturbance during early development [248]. For example, contactin-associated protein-2 (CASPR2) is identified as a neuronal-surface target for anti-brain antibodies from the mothers of children with ASD [247]. Intrauterine exposure to CASPR2–IgGs leads to persistent microglial activation concurrent with abnormal location and synaptic loss of glutamatergic neurons in the somatosensory cortex, which contributes to the social deficits in adulthood [249]. Work from Rosin and colleagues demonstrated that embryotic hypothalamus is comprised of microglia that segregate into four subtypes distinct by unique gene signature, in which the Spp1^+^ population located along the third ventricle directly contact with nearby neural stem cells (NSCs). Maternal cold stress elevates the secretion of CCL3 and CCL4 from Spp1^+^ microglia, which alters the proliferation and differentiation of NSCs and decreases oxytocin neurons in paraventricular nucleus that associated with social behaviors [250]. In addition, patients with tuberous sclerosis (TSC), a genetic disorder with mutations in the *Tsc1* or *Tsc2*, have a high prevalence of ASD. In *Tsc2*^*−/−*^ mice undergoing early postnatal immune activation, social memory deficit correlates with a long-lasting upregulation of mTOR/IFN-β pathway in microglia, consistent with the observations in immune activated mouse model of microglia-restricted *Tsc2* mutation, revealing the centrality of microglia in the synergistic interactions between early immune activation and genetic mutations associated with ASD [251].

Of note, the immune perturbations during pregnancy continuously impact microglia–synapse interaction in the offspring throughout lifetime. At both E17 and P60, MIA microglia exhibit upregulation of cellular protrusion/synaptogenic factors, such as catenin delta 2 (*CTNND2*), neuronal cell adhesion molecule 2 (*NCAM2*) and neurotrophic receptor tyrosine kinase 2 (*NTRK2*), suggesting that MIA drives lifelong disruption of microglial transcriptome towards an enhanced synaptogenic state. The aberrant microglial function coalesces with immature synaptic surplus of intrinsically bursting neurons in the mPFC and enhanced autistic-like behaviors in the MIA offspring, which can be reversed by microglia repopulation using CSF1R inhibitor [252]. In developing brain, deficient microglial elimination of hippocampal mossy fiber synapses is observed in MIA offspring with ASD phenotype. Moreover, voluntary exercise in adulthood ameliorates their synaptic and behavioral abnormalities by activating a portion of dentate granule cells that primes microglia to engulf weaker synapses [253]. Taken together, microglia sensing and responses to fetal stress put them in the unique position linking MIA with the susceptibility to ASD [247].

Dysregulation of protein synthesis is one of the recognized biochemical pathways underlying ASD pathophysiology. Mutations of several translation regulators, including eukaryotic initiation factor 4F (eIF4E), eIF4G and some upstream negative regulators of mTORC1 (PTEN, TSC1 and TSC2), have been reported associated with ASD [254–256]. The mechanistic underpinnings of these mutations remain largely unknown. Xu and colleagues elevated mRNA translation by overexpressing eIF4E and found that exaggerated protein synthesis in microglia, but not neurons, led to social deficit, repetitive behaviors, and cognitive defect in male mice. The authors further showed the decreased mobility of these microglia prevented them from clearing the synaptic surplus, which resulted in increased synapse density and excitation/inhibition (E/I) ratio in the cortex [257]. These findings indicate that microglia with abnormal protein metabolism could be the initiator indispensable to the onset of some ASD-related behaviors.

### Schizophrenia

Schizophrenia is a psychiatric syndrome characterized by delusions, hallucinations, and negative symptoms, such as diminished motivation and expressiveness concomitant with cognitive deficits [258]. According to the unified gliocentric model of schizophrenia proposed by Dietz et al., microglia might be the key mediator between developmental risk factors (e.g., MIA and emotional maternal stress) and the vulnerability to schizophrenia. Early immune activation of microglia during brain development suppresses the proliferation and differentiation of glial progenitor cells, leading to delayed and deficient maturation of astrocytes and oligodendrocytes. Consequently, these glial impairments result in the disruption of white matter integrity and microenvironmental homeostasis in CNS, causing cerebral desynchronization and dysconnectivity [259].

MIA is a contributing factor underlying persistent high neuroimmune status in patients with schizophrenia [260]. Findings from in vitro cultures showed that co-culture with activated microglia compromised the metabolic pathways in developmental cortical interneuron (cIN) rather than the more expected immune disturbances, as indicated by impaired mitochondrial metabolism and synaptic formation. Moreover, after removal of activated microglia-conditioned medium, cINs from schizophrenia donors, but not healthy individuals, exhibited long-lasting metabolic deficits, suggesting an interaction between schizophrenia genetic backgrounds and inflammatory environment induced by microglial activation [261]. In addition, appropriately 25% of the schizophrenia patients have an incidence of catatonia, which might be triggered by activated microglia and low-grade inflammation in the white matter tracts [262].

Postmortem studies demonstrated that loss of dendritic spines in the PFC is a common neuropathological alteration in schizophrenia individuals. C4-mediated excessive synapse clearance is suggested to involve in the pathogenesis of schizophrenia. In individuals, the elevation of C4 level is significantly related to the increased TSPO in the brain [263]. Using schizophrenia–patient-derived neural cultures, Sellgreen and colleagues showed enhanced synapse uptake by microglia-like cells mediated by neuronal C4 deposition [264]. Likewise, C4a overexpression increased microglial pruning of synapses in the mPFC, rendering reduced synaptic density and altered behaviors in adult mice that resemble the negative symptoms experienced by schizophrenia individuals [265]. Given these findings, interventions targeting microglial immune response or synapse elimination merit further research for the prevention or treatment of schizophrenia.

### Fear memory

Fear memory assumes a central role in the onset and development of trauma and stress-related neuropsychiatric disorders, such as post-traumatic stress disorder (PTSD) [266]. Transcriptomic analysis displayed the persistent gene expression programs in microglia supporting the consolidation of remote fear memory, specifically enriched in innate immunity (*Il6r, Il1a, Stat6, Csf3r, CD86*, etc*.*), cytoskeletal rearrangement and adhesion maintenance pathways (Cdc42, Vasp, Rhoa, Rhoh, Prkcd, etc*.*), implying that enhancement of inflammatory response and cell migration might be entangled in the maintenance of fear memory [267]. Nguyen and colleagues revealed that the synapse remodeling associated with memory encoding is governed by neuron–microglia signaling via IL-33. IL-33 is secreted by hippocampal neurons in an experience-dependent manner to prime microglial phagocytosis of extracellular matrix (ECM), and hence the loss of IL-33 results in accumulation of ECM surrounding synapses and decrease in newborn neurons, consequently leading to impaired synaptic plasticity and diminished remote fear memories [129]. In the dentate gyrus, microglia were shown to eliminate the dendritic spines of engram cells via C1q, promoting the disassociation of engrams and erasure of contextual fear memory [268].

### Pain

The International Association for the Study of Pain defines pain as “an unpleasant emotional experience associated with, or resembling that associated with, actual or potential tissue damage”. Physical, mental or emotional pain have expanded the definition of pain and recently all of these have been included in personal emotional experience [269]. It is becoming clear that spinal cord microglia play an critical role in chronic neuropathic pain [270]. In human spinal cord undergoing peripheral nerve injury, there is a large subpopulation of microglia exhibiting a transcriptional profile (*ApoE, Lpl, Spp1, CD6*3 and *CTSB*) that remarkably similar to the previously described injury-responsive microglia (IRM) and proliferating DAM/ATM. Particularly, the upregulation of ApoE might represent a central switcher controlling the transcriptional shift from immunoreactive microglia towards a metabolically altered state [271]. These activated microglia degrade the perineuronal nets (PNNs) that selectively enwrap the spinal parabrachial projection neurons responsible for nociceptive information integration and transduction in the dorsal horn, and thus they can augment the output of spinal nociceptive circuitry [272]. Persistent synaptic potentiation change also contributes to the chronic hyperalgesia, which is triggered by neuronal released neuromodulators (SP, ATP, BDNF, CASP6, or CSF1). Spinal long-term potentiation (LTP) at C-fiber synapses is hypothesized to underlie the chronic pain. High frequency stimulation (HFS) or sciatic nerve injury can induce LTP at the C-fiber and upregulate CSF1 only in calcitonin gene-related peptide (CGRP^+^) terminals and CSF1R on spinal microglia. Subsequently, this CSF1–microglial BDNF signaling increases the number of CGRP terminals in the spinal dorsal horn. CGRP^+^ C-terminals are critically involved in the initiation and maintenance of microglial activation in spinal dorsal horn. In turn, selective deletion of spinal microglia blocks the high-frequency induced LTP and chronic pain hypersensitivity. In this crosstalk, CSF1 and BDNF signaling in microglia are indispensable for spinal LTP and chronic pain [273]. CD11c^+^ microglia seem to exert pleiotropic effects on the neuropathic pain. In nerve-injured mice, CD11c^+^ microglial ablation hinders the recovery from pain hypersensitivity, whereas pain-recovered mice with depletion of CD11c^+^ microglia relapses in hyperalgesia. The authors further defined IGF1 signaling of CD11c^+^ microglia as the key regulator in the dynamic equilibrium between ongoing pain facilitation and repression, which might be the determinant for the remission and recurrence of pain [274]. Given the heterogeneity of microglia in neuropathic pain, further studies will apply microglial manipulation tools, including chemo- or optogenetic selective activation, to investigate their function in pain, aversion, and comorbidities during chronic pain diseases [275].

### Compulsive or feeding disorders

**Fig. 2:**
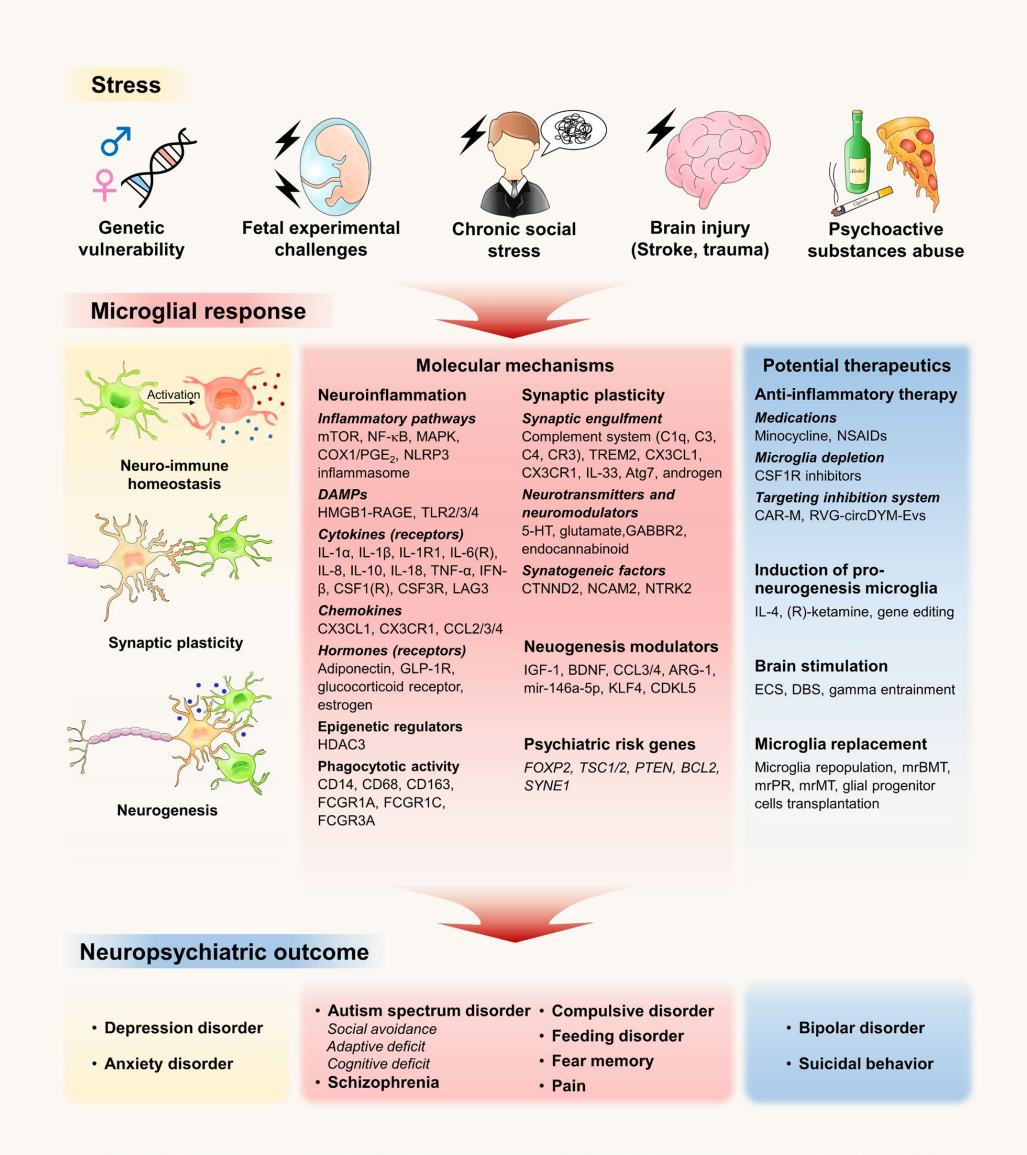
Microglial contribution to neuropsychiatric disorders. Since embryonic period, biological and social stress hijack microglia to dysfunctional states that trigger neuroinflammation, synaptic abnormality, and diminished neurogenesis in various brain areas responsible for emotional and behavioral regulation, which consequently predispose individuals to the onset or deterioration of neuropsychiatric disorders. Further understanding of microglia will shed light on the microglia-targeted therapeutics for more efficient and personalized treatment of neuropsychiatric disorders

Feeding and eating disorders include anorexia nervosa, bulimia nervosa, binge eating disorder, avoidant-restrictive food intake disorder, pica and rumination disorder, which are often companied with obsessive–compulsive or autism spectrum traits [276]. Within the CNS, hypothalamus controls energy metabolism but also directly senses and responds to peripheral hormones and glycolipid metabolites [277–279]. The interaction between neurons and microglia in hypothalamus has been shown to be engage with body weight homeostasis and diet-induced obesity (DIO) [280]. UCP2 is highly expressed in activated microglial cells in DIO accompanied by abnormal mitochondrial morphology. Microglial UCP2 deficiency prevents mitochondrial morphological alterations and dysfunction in the arcuate nucleus induced by high-fat diet (HFD), ultimately decreasing the susceptibility to obesity by remodeling the synaptic input organization and anorexigenic hypothalamic POMC neurons [281]. Besides, Cheng et al. reported that HFD consumption impedes threat-cue-induced suppression of compulsive sucrose-seeking behavior in mice. This compulsive behavior was attributed to enhanced cue-triggered neuronal activity in the anterior paraventricular thalamus (aPVT) due to HFD-induced microglia activation [282] (see Fig. 2).

## Future perspectives for the field

### Sex bias: can microglia explain gender differences of neuropsychiatric disorders?

Sexual dimorphisms prevail in depression [202], anxiety [283], ASD [284] and schizophrenia [285]. Biological mechanisms including neuroinflammation [283], biochemical reactions [257], and more delicate transcriptional and proteomic divergence [286, 287] may underlie the pathophysiology differing between males and females; meanwhile, microglia may play a mediating role in these dimorphisms. Microglial differentiation and responses to environmental challenges diverge in males and females. They remain sensitive to sexual hormones throughout lifespan and modify sex-specific circuits in the preoptic area, cerebellum and amygdala [4, 288, 289]. The pro-inflammatory microglia of depressive-like phenotypes (PIMID), as introduced before, was found in female cynomolgus macaques that more vulnerable to social stress-associated depression than males [214]. In male developmental amygdala, microglia are more phagocytotic regulated by the androgen-induced endocannabinoid (eCB) tone and thereby shape the male social circuitry [290]. Elevating microglial protein synthesis only induced impaired synaptic refinement and autism-like behaviors in male but not female mice [257]. Microglia appear to be more vulnerable in male embryos and female adults, which may underly the higher incidence of ASD and schizophrenia in males at early stage, yet depression are more prevalent in females during adolescence or adulthood [4, 291–293].

### Species diversity: how are human microglia involved in neuropsychiatric pathology?

Although considerable animal experiments have deciphered the involvement of microglia in various neuropsychiatric disorders, the confirmations in humans are still sparse. As discussed above, TSPO–PET and post-mortem studies of MDD patients remain controversial on whether microglia are activated as observed in most rodent models. The cellular repertoire in primate dorsolateral prefrontal cortex revealed the expression of neuropsychiatric risk gene FOXP2 in human-specific microglia, representing an evolutionary specialization of human microglia associated with psychiatric diseases [294]. Genome-wide association studies (GWASs) of human brain endorsed disease-specific alterations in microglia associated with ASD, schizophrenia and bipolar disorder, predominantly enriched in immune responses and phagocytosis [200, 201, 295]. Given the high inter-individual variations of neuropsychiatric disorders, large-scale clinical studies with more selective inclusion criteria are warranted to provide more compelling evidence. Of note, since the understanding of microglial diversity has been far beyond M1/M2 polarization, and their functional repertoire has immensely expanded over immunoreactivity and phagocytosis, whether microglia are involved in neuropsychiatric pathology should probably not be concluded by conventional inflammatory or phagocytotic markers. For this reason, confirming human microglia diversity and reliable markers at specific disease contexts could be an urgent problem.

Indeed, the gene expression profiles of human microglia are not identical to the data of rodents, showing differences in genes associated with immune regulation, proliferation and cell cycle [8]. Several researches revealed the unique spatiotemporal transcriptomics of human microglia obtained from individuals with aging, neuroinflammatory or neurodegenerative diseases [8, 26]. Furthermore, studies of live microglia behavior from fresh brain samples have extended the understanding of human microglial dynamics interacting with neurons, glial cells and multiple molecular signals within the pathological microenvironment. For example, work in human epileptic tissues exhibited distinct microglial live responses to purinergic stimulation in disease states, including membrane ruffling and process extension or retraction [296, 297]. The sophisticated combination of live imaging and human microglia models constructed by ex vivo acute brain slices, in vitro human-induced pluripotent cells or embryonic stem cells, brain organoids, and even in vivo human brain organoids using xenotransplantation [298–301], will allow neurologists towards better comprehension of human microglia pathology.

### Bench to clinic: are microglia new targets for neuropsychiatric disorder treatment?

Although human microglia biology remains largely unknown, the prospect of targeting microglia for neuropsychiatric disorders treatment is fascinating. Current promising directions include:

#### Anti-inflammatory therapy

Microglia deletion (e.g., using CSF1R inhibitor) or inhibition (e.g., using minocycline) appears to mitigate neuroinflammation, emotional and behavioral disorders in animal models [219, 302–305]. In clinical trials, although minocycline seem not to improve treatment-resistant depression [306], schizophrenia [307, 308] and bipolar disorder [304], its add-on efficacy was demonstrated in MDD patients with low-grade peripheral inflammation (CRP ≥ 3 mg/L) [309]. More innovative microglia-targeted systems, such as a melatonin releasing system using cytotoxic T-lymphocyte-associated protein-4 (CTLA-4) as a chimeric antigen receptor (CAR) to target CD86 on microglial surface (CAR-M) [310], and circDYM (mm9_circ_0007509) transfer system via extracellular vesicles (EVs) modified by acetylcholine receptor‐specific rabies virus glycoprotein (RVG-circDYM-EVs) [311], both of them can enter the brain across BBB, suppress microglial inflammatory activation, and alleviate depressive-like behaviors.

#### Induction of pro-neurogenesis microglia

Medications such as IL-4 [55] and (R)-ketamine [312] demonstrate antidepressant effects by reprograming microglia towards an ARG-1 expressing phenotype that promotes BDNF-dependent neurogenesis and rescues synaptic loss. Since activated microglia was found in the vicinity of new-born neurons to comprise their survival, microglia inhibition by minocycline may also have a pro-neurogenesis effect [305, 313].

#### Brain stimulation

Methods include transcranial magnetic stimulation, transcranial direct current stimulation, electroconvulsive stimulation (ECS) and deep brain stimulation [314]. Microglia appear to be necessary for the antidepressant effect of ECS, during which ECS can inhibit the immune checkpoint gene lymphocyte-activating gene-3 (*LAG3*) in microglia and enhance hippocampal neurogenesis [315]. Gamma entrainment has been investigated in treatment of various CNS diseases [316, 317]. In AD models, gamma entrainment enhanced microglial phagocytotic activity [318, 319]; in post-stroke models, gamma entrainment inhibited the pro-inflammatory activation of microglia and ameliorated anxiety susceptibility [207]. However, there is emerging controversy about the neuroprotective effect of gamma entrainment, and the gamma-band visual stimulation may be aversive for mice [320].

#### Cell replacement therapy

With the understanding of microglia repopulating properties, microglia replacement by pharmacologically induced repopulation, bone-marrow transplantation (mrBMT), peripheral blood (mrPB), or microglia transplantation (mrMT) have been explored as novel therapeutics [321–323]. The most recent work by Vieira et al*.* engrafted human glial progenitor cells (hGPCs) into mice chimerized with mutant Huntingtin (mHTT) expressing hGPCs, which archived effective repopulation with younger healthy glia that broadly replaced aged and diseased human glia, representing a markable progress of microglia replacement as a therapeutic strategy for CNS diseases [324].

In summary, high-throughput sequencing has unveiled the temporal–spatial heterogeneity of microglia about their ontogeny, phenotype diversity and biological functions. Pathophysiology of neuropsychiatric disorders is an intricate combination of genetic risk, biological stress, social threats, sex, age, comorbidity with other physical or psychiatric disorders, etc*.*, and the versatile microglia may be involved in each catalyst. A deep comprehension of microglia biology will provide window into the microgliopathy of neuropsychiatric disorders, exemplified by the microglia supporting neurogenesis in depression, synaptic elimination in ASD and schizophrenia, microglia–neuron crosstalk in eating disorders and other to-be-explored mechanisms. Most importantly, these discoveries need to be confirmed in humans to break the bottleneck of neuropsychiatric disorders treatment. In the future, multiple advanced technologies and high-quality clinical researches will shed light on the shadow of microglia casting over human brain diseases.

## Data Availability

Not applicable.

## Abbreviations

Aβ: Amyloid β
AD: Alzheimer’s disease
ALS: Amyotrophic lateral sclerosis
ApoE: Apolipoprotein E
ATM: Axon tract-associated microglia
ATP: Adenosine triphosphate
α-syn: α-Synuclein
ACC: Anterior cingulate cortex
ASD: Autistic spectrum disorder
BBB: Blood–brain barrier
BDNF: Brain-derived neurotrophic factor
AMP: Cyclic adenosine monophosphate
CARD9: Domain-containing protein 9
CNS: Central venous system
CSF1R: Colony stimulating factor 1 receptor
CCR5: C–C chemokine receptor type 5
CCRL2: C–C motif chemokine receptor-like 2
CX3CR1: C–X3–C motif chemokine receptor 1
CXCR4: C–X–C chemokine receptor type 4
CXCL1: C–X–C motif chemokine ligand 10
CAM: Capillary-associated microglia
CLDN5: Claudin-5
CASPR2: Contactin-associated protein-2
cIN: Cortical interneuron
CGRP: Alcitonin gene-related peptide
DAM: Disease-associated microglia
DIO: Diet-induced obesity
ECM: Extracellular matrix
eIF4E: Eukaryotic initiation factor 4F
FGF2: Fibroblast growth factor 2
GVU: Glial–vascular unit
GLP-1R: Glucagon-like peptide-1 receptor
HDAC: Histone deacetylase
HFD: High-fat diet
Iba-1: Ionized calcium-binding adaptor molecule 1
iNOS: Inducible nitric oxide synthase
IGF-1: Insulin-like growth factor 1
IFN: Interferon
IL: Interleukin
IFP35: Interferon-induced protein 35
IRM: Injury-responsive microglia
LDAM: Lipid droplet accumulating microglia
LPS: Lipopolysaccharide
MGnD: Microglial neurodegenerative phenotype
MS: Multiple sclerosis
MMP-9: Metalloproteinase 9
MIMS: Microglia inflamed in MS
MIA: Maternal immune activation
mrBMT: Microglia replacement by bone-marrow transplantation
mrPB: Microglia replacement by peripheral blood
mrMT: Microglia replacement by microglia transplantation
mPFC: Medial prefrontal cortex
MDD: Major depressive disorder
NF-κB: Nuclear factor kappa-B
NLRP3: Nucleotide-binding oligomerization domain
NLRP3: NOD-like receptor family pyrin domain-containing 3
NVU: Neurovascular unit
OB: Olfactory bulb
OCD: Obsessive–compulsive disorder
OPCs: Oligodendrocyte precursor cells
PAM: Proliferative region-associated microglia
P2RY12: Purinergic receptor P2Y12
PtdSer: Phosphatidylserine
PDGFB: Platelet-derived growth factor B
PTSD: Post-traumatic stress disorder
PNNs: Perineuronal nets
RIPK1: Receptor interacting protein kinase 1
ROS: Reactive oxygen species
RRIMs: Regulated inflammatory microglia
R-SDS: Repeated social defeat stress
Spp1: Secreted phosphoprotein 1
SVZ: Subventricular zone
SYK: Spleen tyrosine kinase
ScRNA-seq: Single-cell RNA-sequencing
SCI: Spinal cord injury
TNF: Tumor necrosis factor
TREM2: Triggering receptor expressed on myeloid cells 2
TLR: Toll-like receptors
TGF-β: Transforming growth factor-β
THIK-1: TWIK-related Halothane-inhibited K + channel
TREM2: Triggering receptor expressed on myeloid cells 2
TRPV1: Transient receptor potential vanilloid type
TSPO: Translocator protein
TSC: Tuberous sclerosis
UCP2: Uncoupling protein 2
VEGFA: Vascular endothelial growth factor A
